# Surgical management of complex congenital heart diseases in the context of South-South cooperation: case series

**DOI:** 10.11604/pamj.2026.53.21.49748

**Published:** 2026-01-16

**Authors:** Laurence Carole Ngo Yon, Hermann Nestor Tsague Kengni, Godwin Sharau, Deogratias Nkya, Stella Mongella, Flora Fondjo, Alex Loth, Vivienne Mlawi, Salvatore Agati

**Affiliations:** 1 Department of Surgery and Specialties, Faculty of Medicine and Biomedical Sciences, University of Yaounde I, Yaounde, Cameroon,; 2 Department of Thoracic and Cardiovascular Surgery, Yaounde General Hospital, Yaounde, Cameroon,; 3 Department of Clinical Sciences, Faculty of Medicine and Biomedical Sciences, University of Yaounde I, Yaounde, Cameroon,; 4 Jakaya Kikwete Cardiac Institute, Dar es Salaam, United Republic of Tanzania,; 5 Department of Health Sciences, School of Health Sciences, Catholic University of Central Africa, Yaounde, Cameroon,; 6 Mediterranean Pediatric Cardiac Surgery Center-Bambino Gesù, San Vincenzo Hospital in Taormina, Taormina, Italy

**Keywords:** Common arterial trunk, aortopulmonary window, interrupted aortic arch, pulmonary hypertension, South-South cooperation

## Abstract

Complex congenital heart diseases, such as common arterial trunk (CAT) and aortopulmonary window (APW) with interrupted aortic arch (IAA), present challenges in low-resource settings where technical limitations restrict surgical care. We describe three cases referred from Cameroon and treated at the Jakaya Kikwete Cardiac Institute (JKCI) in Tanzania through authentic South-South cooperation with the purpose of describing clinical, anatomical, and surgical aspects of these patients, and assessing this collaborative model. The patients, aged 7 months to 3 years, included two CATs and one APW with an interrupted aortic arch, all complicated by severe pulmonary hypertension. Multidisciplinary management allowed for surgical repair in two of the cases, but one child died intraoperatively despite intensive intervention, while the third was deemed inoperable and remained on medical management. These cases are illustrative of the viability and benefits of South-South cooperation, of the role of multidisciplinary care and building capacity locally for improved outcomes.

## Introduction

Complex congenital heart diseases, such as aortopulmonary window (APW) and common arterial trunk (CAT) with interrupted aortic arch (IAA), have emerged as a major public health issue in resource-limited countries. Their management requires highly specialised expertise, advanced and adequate technology, and a multidisciplinary approach [1], which are often lacking in African healthcare systems, especially in sub-Saharan Africa [2].

Historically, systematic medical evacuations to high-income countries have long been seen as the only viable alternative [3]. However, this option has shown clear limitations, mainly due to high costs and difficult administrative procedures. In this context, South-South cooperation in healthcare has emerged as a strategic, innovative, and solidarity-based alternative, tailored to local realities, enabling the development of local expertise and the strengthening of healthcare resources through shared experiences among countries within the same continent [4,5].

We report herein three cases of complex congenital heart disease, two cases of CAT and one case of APW associated with IAA, surgically managed as part of a medical evacuation from Cameroon to a pediatric cardiac surgery referral centre in Tanzania. Through this experience, we aim to highlight not only the clinical and surgical considerations of these rare conditions but also the potential benefits of South-South cooperation for patients, healthcare providers, and health systems in Africa.

## Methods

**Study design and setting:** this study was a descriptive retrospective case series involving pediatric patients with complex congenital cardiac defects, and it aimed to describe the clinical presentation, diagnostic evaluation, decision-making, operative management and outcomes within a South-South collaborative program. It was a collaborative initiative between the Cardiac Surgery Unit of the Yaoundé General Hospital (*Hôpital Général de Yaoundé*-HGY) in Cameroon and the Jakaya Kikwete Cardiac Institute (JKCI) in Dar es Salaam, Tanzania. HGY is a tertiary referral hospital for patients with cardiac disease in Cameroon, while JKCI is a regional specialised hospital providing advanced pediatric cardiac surgical management for patients from East Africa. All surgical procedures were performed at JKCI in April 2025.

**Participants:** patients were considered for inclusion in the study if they had been diagnosed with a complex congenital cardiac defect that requires specialised surgical management not available in Cameroon. The subject was referred from HGY to JKCI during the study timeframe and had been assessed with full clinical, imaging, surgical, and post-operative data. A total of three pediatric patients met the above inclusion criteria and were therefore included in this study.

**Variables:** they included socio-demographic information, diagnostic imaging data: transthoracic echocardiography, computed tomography (CT) angiography with 3D reconstruction, and, where performed, cardiac catheterisation parameters that captured pulmonary-to-systemic flow ratio (Qp/Qs) and pulmonary vascular resistance, clinical data, intraoperative data and postoperative outcomes.

**Data sources/measurement:** all the data were retrieved through retrospective review of hospital records from HGY and JKCI. Imaging studies were reviewed from institutional archives, and all intraoperative details were reviewed from surgical reports.

**Bias:** selection bias was minimised by including all consecutive patients who met the predefined inclusion criteria during the study period. The retrospective nature of the study might limit the completeness of some clinical data.

**Size of study:** this study included three patients who were pediatric patients, and they corresponded to all eligible cases referred from HGY to JKCI for advanced surgical management of complex congenital cardiac defects within the study window.

**Statistical methods:** given the small sample size and descriptive nature of the study, no statistical comparisons or inferential analyses were performed. Data were summarised narratively and presented in tabular form.

**Ethical considerations:** our institution does not require ethical approval for reporting individual cases or case series. Written informed consent was obtained from a legally authorised representative for anonymised patient information to be published in this article. This case series was conducted in accordance with the ethical principles outlined in the Declaration of Helsinki [6].

## Results

**Participants:** three pediatric patients with complex congenital cardiac defects referred from the Cardiac Surgery Unit of the HGY, Cameroon, to the JKCI, Tanzania, were included in this case series. They ranged in age from 7 months to 3 years, comprising two girls and one boy. All patients presented with signs of heart failure, growth retardation, and NYHA functional class II symptoms.

### Descriptive and outcome data

**Figure 1:**
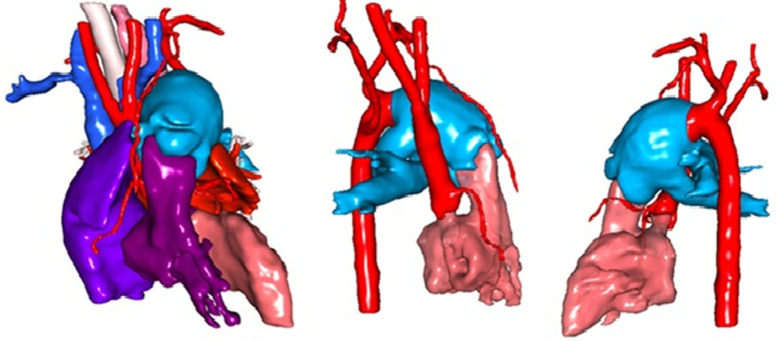
3D reconstruction of computed tomography images showing a type II truncus arteriosus according to Collett and Edwards

**Case 1:** a 3-year-old girl was referred to HGY, Cameroon, for CAT with IAA. She exhibited moderate heart failure with feeding difficulty, exertional corresponding to NYHA II, tachypnea, sweating, intercostal retraction, delay in growth, and clubbing. On examination, there were 90% oxygen saturations, suprasternal thrill, a grade 3/6 systolic murmur at the pulmonary area, well-palpated peripheral pulses and preserved psychomotor development. Echocardiography and CT angiography confirmed type II truncus arteriosus (22 x 24 mm) according to the Collett and Edwards classification, overriding a 15 mm ventricular septal defect (VSD), with a competent tricuspid truncal valve; separate origin of the pulmonary arteries with right posterior and left postero-lateral. A 3D CT reconstruction is shown in Figure 1. Other findings included right ventricular hypertrophy, severe enlargement of the right atrium, pulmonary arterial hypertension (PAH), retro-oesophageal right subclavian artery compressing the oesophagus, and diffuse pulmonary hypervascularization.

At JKCI, echocardiography confirmed type II truncus arteriosus. Due to advanced age, complex anatomy, chronic PAH, and overall fragility, the multidisciplinary team opted for optimised medical therapy as opposed to surgery. The patient was discharged to Cameroon in a clinically stable condition.

**Figure 2:**
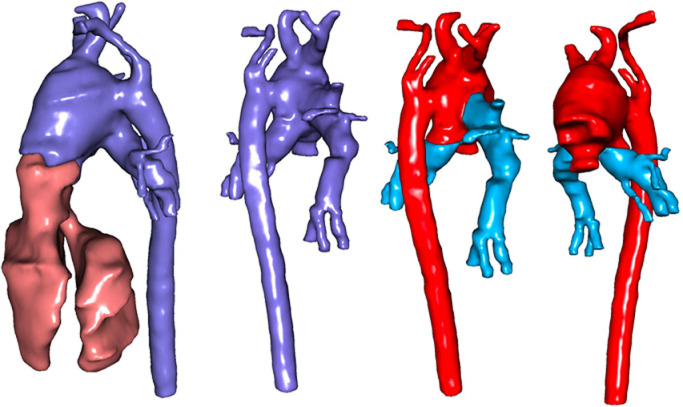
3D reconstruction of computed tomography images showing a type A4 truncus arteriosus according to Van Praagh

**Case 2:** a 7-month-old male infant was referred to HGY with CAT and IAA, presenting with heart failure, dyspnea, NYHA II, tachypnea, cyanosis, retractions, and growth delay. Oxygen saturation was 80%, with a 3/6 systolic murmur; psychomotor development was preserved. CT angiography confirmed type A4 truncus according to Van Praagh *et al*. [7] overriding a VSD with a quadricuspid truncal valve, separate origins of the pulmonary arteries, and IAA. A 3D CT reconstruction is shown in Figure 2.

At JKCI, echocardiography confirmed type A4 truncus with dysplastic truncal valve regurgitation, interrupted arch, and severe PAH. Catheterisation showed Qp/Qs >3 and pulmonary vascular resistance improving from 9.49 to 5.07 Wood units with 100% oxygen. A multidisciplinary team deemed operative risk very high but, after discussion and parental consent, proceeded with complete repair under general anaesthesia with full monitoring.

*Surgical technique:* a median sternotomy with thymectomy and pericardiotomy exposed a type A4 truncus arteriosus according to Van Praagh *et al*. [7] giving rise to separate pulmonary arteries of approximately 10 mm in diameter and an IAA, with the brachiocephalic and left carotid arteries from the truncus, and the left subclavian from the descending aorta. Surgery involved mobilisation of the truncus, aortic arch, and pulmonary arteries, followed by CPB via aorto-bicaval cannulation and cooling to 25°C. The pulmonary arteries were dissected free, temporarily ligated, and the aorta was cross-clamped. After cold Del Nido cardioplegia, following right atriotomy, a 4 mm interatrial communication was created. The truncus was divided, reshaped, and excess tissue resected. A right ventriculotomy allowed VSD closure with a bovine pericardial patch, ensuring unobstructed LV outflow. A 16 mm Gore-Tex conduit connected the RV to pulmonary arteries, and the neo-aorta was reconstructed by end-to-end anastomosis.

The patient was gradually rewarmed. The atriotomy was closed. Cardiac chambers were meticulously deaired, and the aortic cross clamp was released, restoring sinus rhythm. Following complete rewarming, attempts to wean from CPB were impeded by recurrent severe pulmonary hypertensive crises. Despite exhaustive resuscitative measures and maximal use of available resources, the patient developed irreversible circulatory arrest, culminating in intraoperative death.

**Figure 3:**
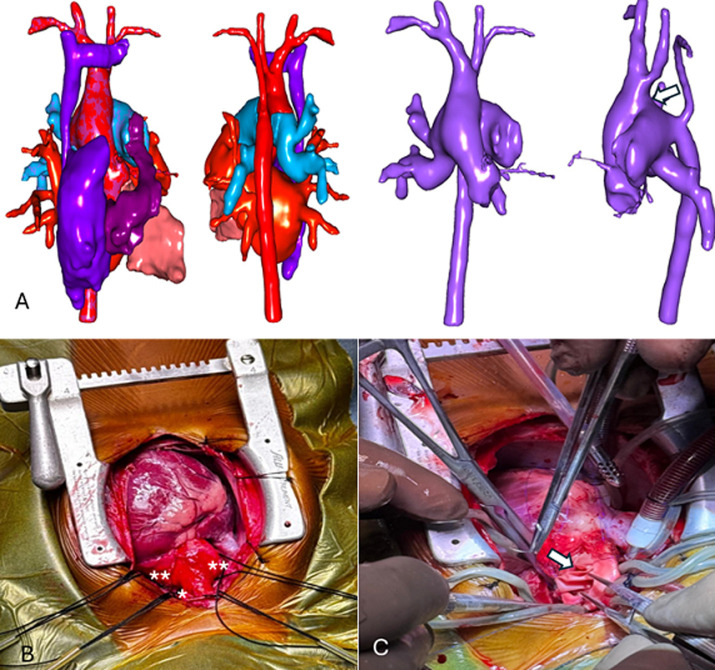
A) 3D reconstruction of computed tomography of the aortic arch and pulmonary arteries, the arrow indicates the aortopulmonary window; B) intraoperative view of the heart, highlighting the major anomalies and mobilised vessels, indicates the isolated ductus arteriosus, showing the right and left pulmonary arteries; C) intraoperative view of the heart, the arrow indicates the reconstruction of the posterior wall of the ascending aorta to the proximal portion of the descending aorta using a bovine pericardial patch

**Case 3:** a 10-month-old girl was referred to HGY with heart failure due to a 10 mm APW with a left-to-right shunt and IAA. A 3D CT reconstruction is shown in Figure 3A. She presented with feeding difficulty, dyspnea NYHA II, tachypnea, sweating, intercostal retractions, and growth delay. Examination showed 90% oxygen saturation, suprasternal thrill, and a grade 3/6 systolic murmur, with preserved psychomotor development. At JKCI, echocardiography confirmed a hemitruncus with large APW type III according to Richardson *et al*. type B interrupted arch, patent ductus arteriosus, right pulmonary artery from the ascending aorta, and severe PAH. Cardiac catheterisation showed Qp/Qs of 1.81 and pulmonary vascular resistance improving from 14.97 to 4.57 Wood units with 100% oxygen. A multidisciplinary team deemed operative risk very high but, after discussion and parental consent, proceeded with surgery under general anaesthesia with full monitoring.

*Surgical technique:* a median sternotomy with thymectomy and pericardiotomy revealed major malformations (Figure 3B) a large APW (15 × 10 mm) extending to the right pulmonary artery, dilation of both pulmonary arteries, IAA, and a 5 mm ductus arteriosus. The pulmonary arteries, ascending aorta, and supra-aortic trunks were mobilised and looped, followed by CPB via aorto-bicaval cannulation and cooling to 25 °C, and Del Nido cardioplegia was administered. After circulatory arrest and antegrade cerebral perfusion, the descending aorta was clamped. The ascending aorta was separated from the pulmonary arteries, and the APW was closed with a bovine pericardial patch to reconstruct the pulmonary artery. The aortic arch was repaired by end-to-end anastomosis anteriorly and posterior wall augmentation with a bovine patch (Figure 3C). After de-airing, reperfusion, and rewarming, the final reconstruction was achieved. A right atriotomy was performed to create a 4 mm interatrial communication for postoperative left heart decompression, which was then closed with two layers of continuous sutures. The heart regained sinus rhythm, the patient was weaned from CPB without incident, drains and pacing wires were placed, and the chest was closed.

*Postoperative course:* the postoperative course required a 32-day intensive care stay due to recurrent pulmonary hypertensive crises and Klebsiella septicemia, managed with antibiotics. On day 33, the patient was transferred to the Cardiology Unit, and by day 40, discharged to Cameroon in good condition on oral furosemide, spironolactone, and sildenafil. The pre-discharge ECG showed sinus rhythm, and echocardiography confirmed successful repair of the aortopulmonary window and aortic arch, though pulmonary hypertension persisted.

**Other analyses:** due to the small sample size and observational design, formal statistical analyses or comparisons were not feasible.

## Discussion

The management of complex congenital heart defects, such as CAT and APW associated with IAA, remains a major challenge in health systems of resource-limited countries. The natural prognosis is well documented to be poor, with mortality rates exceeding 90% before the age of one year in the absence of surgical intervention [7,8].

CAT is a rare congenital anomaly with an estimated incidence of 3 to 10 per 100,000 live births, accounting for approximately 4% of all critical congenital heart defects [9], classified by Van Praagh *et al*. [7] or Collett-Edwards systems. According to Van Praagh *et al*. [7], CAT is divided into types A and B based on the presence (type A) or absence (type B) of a ventricular septal defect, with a further four subdivisions based on the anatomical arrangement of the great arteries. Type A1 corresponds to an incompletely formed aorticopulmonary septum with a partially separate main pulmonary artery. In type A2, the septum is absent, and both pulmonary arteries arise separately from the truncus. Type A3 involves the absence of one pulmonary artery with collateral supply, while type A4 is associated with interruption, hypoplasia, or coarctation of the aortic arch and a large ductus arteriosus. Collett and Edwards [10] proposed another classification, identifying four types of CAT. In type I, a single pulmonary trunk and the ascending aorta arise from the truncus. In type II, the two pulmonary arteries arise close together from the posterior aspect of the truncus. In type III, the pulmonary arteries arise separately from either side of the truncus. Type IV refers to absent pulmonary arteries, with pulmonary blood flow supplied via major aortopulmonary collaterals. CAT is also associated with a single truncal valve, usually tricuspid but occasionally quadricuspid or even up to six cusps [8,10,11], and may present with dysplasia or regurgitation [12]. Our first case was type II CAT according to Collett and Edwards [10], while the second was type A4 according to Van Praagh *et al*. [7].

APW is another rare congenital anomaly, accounting for only 0.2 to 0.6% of congenital heart defects [13,14], usually proximal or distal, but may involve abnormal pulmonary artery origin. Mori *et al*. classified APW into three types: Type I, proximal, near the semilunar valves; Type II, distal, near the bifurcation of the pulmonary arteries; and Type III, a large defect involving nearly no separation between the aorta and the pulmonary trunk [15]. Richardson *et al*. later proposed a similar system, but in their classification, Type III described the abnormal origin of a pulmonary artery directly from the ascending aorta, sometimes misidentified as a hemitruncus [16]. Our third case combined a type III APW with type B IAA.

APW causes a significant left-to-right shunt and elevated pulmonary blood flow, rapidly leading to PAH as in our third case. Without early surgical repair, there is a high risk of developing Eisenmenger syndrome, with reversal of the shunt and central cyanosis. Mortality is estimated to be between 40-50% within the first year of life without surgery [17]. Early surgical closure, ideally before six months of age, has been shown to improve survival and reduce the long-term impact of PAH.

Beyond individual cases, South-South collaborations, as exemplified by the JKCI, demonstrate that complex congenital heart surgery can be achieved in Africa with growing autonomy [18]. Centres such as JKCI and the Salam Centre in Sudan show that sustainable partnerships, targeted training, and regional networks improve outcomes, reduce dependence on foreign transfers, and strengthen local capacity for high-quality pediatric cardiac care [19].

**Limitations:** this case series has some limitations. The first limitation is that the small number of patients limits the applicability of our findings and does not allow statistical analysis. Second, the retrospective way the data was collected may carry a risk of incomplete information or reporting bias. Third, the authors did not have long-term follow-up available for all patients, which limits assessment of late outcomes and the durability of procedures. Last, clinical decisions and operative techniques were influenced by the availability of resources and the experience of the institution, which may differ from that in a high-income setting.

## Conclusion

Complex congenital heart diseases such as CAT and APW with IAA remain a major challenge in resource-limited settings, where prognosis is poor without early surgery. Our three cases highlight the need for multidisciplinary evaluation, family involvement, and timely referral to specialised centres. The partnership between HGY and JKCI illustrates the value of South-South collaboration, enabling high-quality care at reduced cost and delay compared to transfers abroad. Strengthening local capacity, fostering regional networks, and building sustainable partnerships are essential to improve outcomes for children with complex congenital heart disease in sub-Saharan Africa.

### What is known about this topic

- Rare complex congenital heart defects such as truncus arteriosus and aortopulmonary window associated with interrupted aortic arch require early surgical correction in specialised centres;
- In low- and middle-income countries, late presentation is frequent due to delayed diagnosis, financial constraints, and limited access to cardiac surgery, leading to poorer outcomes;
- Successful management depends on timely referral to tertiary centres with cardiopulmonary bypass facilities and multidisciplinary pediatric cardiac teams.

### What this study adds

- This case series shows the feasibility and outcomes of managing rare, complex congenital heart defects in a resource-limited African setting through South-South collaboration between Cameroon and Tanzania;
- It illustrates decision-making from optimised medical therapy to surgical correction, based on anatomy, patient age, and pulmonary vascular status;
- It underscores the importance of regional cooperation, multidisciplinary care, and early presentation to improve access to life-saving interventions for children with complex congenital heart disease in sub-Saharan Africa.
